# Dataset of audiovisual unscripted monologues with speech annotations

**DOI:** 10.1016/j.dib.2026.112932

**Published:** 2026-06-04

**Authors:** Manuela Jaeger, Mareike Daeglau, Giso Grimm, Martin G. Bleichner

**Affiliations:** a Neuropsychology Lab, Department of Psychology, Carl von Ossietzky Universität Oldenburg, Oldenburg, Germany; b Department of Medical Physics and Acoustics, Carl von Ossietzky Universität Oldenburg, Oldenburg, Germany; c Translational Psychology, Department of Psychology, Carl von Ossietzky Universität Oldenburg, Oldenburg, Germany; d Research Center for Neurosensory Science, Carl von Ossietzky Universität Oldenburg, Oldenburg, Germany

**Keywords:** Spontaneous speech, German, Linguistic research

## Abstract

This article presents a publicly accessible multimodal dataset comprising audiovisual recordings of unscripted German monologues and a corresponding time-resolved linguistic speech annotation corpus. The audiovisual dataset consists of more than 300 minutes of natural speech material recorded from six speakers across 60 takes. Recordings were captured using a Canon EOS 700D camera and a Neumann KM-184 microphone, with audio sampled at 48 kHz and video at 25 frames per second. Speakers produced spontaneous monologues on self-selected everyday topics under controlled laboratory conditions. Recording sessions included variations such as background babble noise presented via in-ear headphones and optional visual modifications, including the use of glasses or lipstick. Audio and video were acquired simultaneously, synchronized during post-processing, and segmented into individual stories.

The accompanying speech annotation corpus provides detailed linguistic information aligned with the audiovisual material at millisecond resolution. An annotation pipeline combining established tools—OCTRA, G2P, MAUS, PHO2SYL, and the RFTagger—was used to derive orthographic transcripts, canonical phonological representations, phonetic segmentations, syllabification, and part-of-speech tags. Manual correction steps were applied to ensure transcription accuracy and to improve the quality of forced alignment. Each recording is accompanied by an events.tsv file containing time-stamped word, phoneme, and pause annotations; a JSON sidecar describing variable metadata; and a machine-actionable HED-formatted event file to support integration with neuroimaging standards such as BIDS. The structure of the resource follows a consistent file-naming scheme to ensure reliable linkage between audiovisual recordings and speech annotations.

This combined audiovisual and linguistic resource supports a wide range of reuse applications, including acoustic-phonetic analysis, linguistic and neurolinguistic research, annotation benchmarking, and the development or evaluation of speech-processing tools. The resource’s naturalistic content and high temporal precision enable detailed examination of spontaneous speech and facilitate replication-oriented research across disciplines.

Specifications Table

**Table:** 

Subject	Computer Sciences
Specific subject area	The dataset contains audiovisual unscripted monologues and an annotated speech corpus for acoustic and linguistic research.
Type of data	Audiovisual recordings (.mp4): Audio has a sampling rate of 48 kHz, audio bitrate of 174 kb/s, Video has a frame rate of 25 fps, video bitrate of 11.3 Mbps Tables (*_events.tsv): subject- and take-specific event spreadsheets containing time-resolved linguistic annotations Text (*_events.json): subject- and take-specific sidecar files describing categorical and value columns of the event spreadsheet Tables (*_HED.tsv): subject- and take-specific event spreadsheets containing time-resolved linguistic annotations in machine-actionable HED format (Hierarchical Event Descriptors)
Data collection	Audiovisual recordings were obtained by using a Canon “EOS 700D” (Firmware version 1.1.4) and a microphone “Neumann KM-184” close to the speaker. Subjects spoke freely on a self-chosen topic. The camera was set up such that subjects face the camera. In the video, face and shoulders are visible and the face is in the center of the video. Audio and video were recorded simultaneously and synchronized using DaVinci Resolve. Audio signals were processed offline to derive speech annotations.
Data source location	Audiovisual recordings were collected in-person in the Gesture Lab (Building W30) at Carl von Ossietzky Universität (Oldenburg, Lower Saxony, Germany). Speech annotations were derived based on the audio signals of these recordings in the Neurophysiology of Everyday Life Group (Building A07) at Carl von Ossietzky Universität (Oldenburg, Lower Saxony, Germany).
Data accessibility	**Audiovisual recording dataset:** Repository name: Zenodo (zenodo.org) Data identification number: DOI 10.5281/zenodo.8082844 Direct URL to data: https://zenodo.org/records/8082844 **Speech annotation corpus:** Repository name: Zenodo (zenodo.org) Data identification number: DOI 10.5281/zenodo.17578693 Direct URL to data: https://zenodo.org/records/17578693
Related research article	**Audiovisual recording dataset:** Daeglau M., Otten J., Grimm G., Mirkovic B., Hohmann V. and Debener S. (2025) Neural speech tracking in a virtual acoustic environment: audio-visual benefit for unscripted continuous speech. Front. Hum. Neurosci. 19:1560558. doi: 10.3389/fnhum.2025.1560558 **Speech annotation corpus:** Agmon G., Jaeger M., Magen E., Pinto D., Perelmuter Y., Zion Golumbic E. and Bleichner M.G. Challenges and Methods in Annotating Natural Speech for Neurolinguistic Research. Neurobiol Lang (Camb). 2025 Sep 5;6:nol.a.12. doi: 10.1162/nol.a.12.

## 1 Value of The Data

• The audiovisual recordings represent a unique dataset of unscripted German monologues, in which speakers recount personal everyday-life episodes. The data provide high-quality audio and video captured under controlled laboratory conditions, offering a rare combination of spontaneous speech content with stable recording quality.
• The corresponding speech annotation corpus provides highly time-resolved linguistic information, including phoneme-, syllable-, word-level segmentation and part-of-speech tags. The millisecond-level precision supports detailed acoustic, linguistic, and neurolinguistic analyses that require fine temporal alignment between linguistic units and audiovisual signals.
• The annotated speech corpus fills a gap in currently available German speech corpora, which rarely include natural, spontaneous audiovisual speech with detailed multi-level linguistic annotation. This makes the resource relevant for research on spoken language structure, German language technology, and cross-linguistic comparisons.
• The multimodal nature of the resource enables use across disciplines, including speech science, neurolinguistics, multimodal communication research, gesture studies, computer vision, audiovisual speech processing, and human–computer interaction. The face- and shoulder-centered camera perspective supports research combining speech with facial or visual cues.
• The inclusion of machine-actionable HED (Hierarchical Event Descriptors) annotations enhances interoperability, particularly for researchers working with neuroimaging datasets following BIDS standards. This allows seamless integration with EEG, MEG, or fMRI studies of speech processing and supports reproducible research.

## 2 Background

Spoken language is a cornerstone of human communication, shaping cognition, learning, and social interaction. Unlike written text, natural speech unfolds spontaneously, marked by disfluencies, fillers, repetitions, and flexible syntax — yet it remains remarkably effective for conveying meaning and intent. Understanding how the brain processes such naturally produced speech is critical for uncovering the neural and cognitive mechanisms that support real-world language comprehension.

Numerous German speech corpora have been collected for corpus linguistics, speech technology, and multimodal communication research. The Leibniz-Institut für Deutsche Sprache Database for Spoken German (DGD), for example, provides access to a large collection of spoken-language corpora, including spontaneous conversations, academic speech, and regional varieties. While these resources offer valuable linguistic and multimodal data, most were developed for language documentation, corpus analysis, or engineering applications, and few provide the combination of controlled audiovisual recordings and temporally precise linguistic annotations required for cognitive neuroscience experiments.

This limitation is particularly relevant for neurolinguistic research. Recent methodological advances, including temporal response function (TRF) analyses and other continuous-speech modeling approaches, enable the investigation of neural speech processing using EEG, MEG, and fMRI under naturalistic listening conditions. These approaches require precisely time-aligned annotations of acoustic, phonetic, lexical, prosodic, and discourse-level features, often at millisecond resolution. However, many existing neurolinguistic studies still rely on read or highly curated materials, such as audiobooks or scripted narratives, which lack the variability and contextual richness of spontaneous speech. This limits the ecological validity of findings and their applicability to everyday communication.

To address this gap, we present a German audiovisual corpus of unscripted monologues recorded under controlled laboratory conditions and accompanied by detailed linguistic speech annotation. This resource enables the study of neural speech tracking, audiovisual integration, and spontaneous language processing under more realistic communicative conditions.

## 3 Data Description

The dataset consists of two complementary components: (1) a German audiovisual unscripted monologue dataset [1], and (2) a corresponding time-aligned speech annotation corpus [2]. In the following, *dataset* refers to the overall multimodal resource, while *corpus* refers specifically to the linguistically annotated speech component.

### 3.1 General description

The audiovisual dataset [1] comprises over 300 minutes of unscripted monologue recordings collected from six native German speakers across 60 recording takes. Individual monologues range from approximately 1 to 20 minutes. The recordings were collected under controlled laboratory conditions and include synchronized audio and video signals.

The accompanying speech annotation corpus [2] contains temporally precisely aligned linguistic speech annotations for selected recordings at word- and phoneme-level resolution. Table 1 provides an overview of the number of takes per speaker, recording duration (mm:ss), the overall recording duration per speaker (mm:ss) and recordings included in the annotation corpus [2]. At the time of speech annotation, the full audiovisual dataset was not yet available in its final processed form. Consequently, the annotation corpus was initially created for a subset of recordings available at that stage. Because the generation and manual verification of time-aligned linguistic annotations is highly time-intensive, the remaining recordings were not annotated within the scope of the current release.

**Table 1:** Overview about the number of takes per speaker, the length of the take (mm:ss) and the overall length per speaker (mm:ss). Takes for which speech annotations are provided in the speech annotation corpus are marked in grey.

### 3.2 Annotation scheme

The speech annotation corpus provides time-resolved linguistic annotations with millisecond precision. Each annotated recording contains event annotations at multiple linguistic levels, including pauses, words, and phonemes.

Each annotation file contains the following variables:

The methods used to generate these linguistic features are described in the Experimental Design, Materials and Methods section.

### 3.3 Corpus file-tree structure

Both dataset components follow a consistent file organization to enable direct correspondence between audiovisual recordings and their linguistic annotations. For each subject and take the data are stored in separate files. The file naming convention is as follows:

**Subject_<*subject_ID*>_Take<*take_num*>_<*add_feature*>.<*extension*>**

• ***subject_ID:*** unique integer number for each subject ranging from 1 to 6.
• ***take_num:*** running integer index with leading zeros (01, 02, …) indicating the take number.
• ***add_feature:*** in some takes an additional feature was introduced.
  ○ “MG” indicating when babble noise was played over in-ear phones
  ○ “LS” indicating that the subject wore lipstick during the take
  ○ “BR” indicating that the subject wore glasses during the take
• ***extension:*** taking the value of .mp4, .json or .tsv

**Fig. 1:**
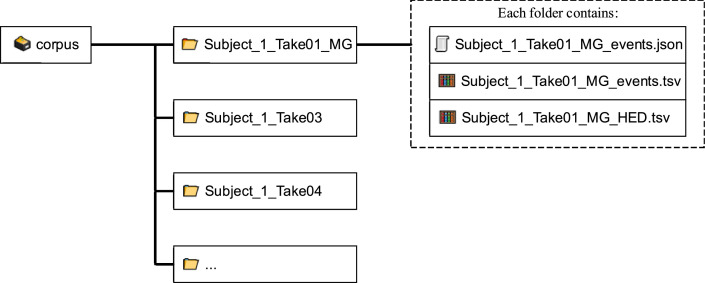
Folder and file structure within the speech annotation corpus. Each subject- and take-level folder contains time-resolved linguistic annotations in *_events.tsv and *_HED.tsv event spreadsheets. The *_events.json sidecar file describes the categorical and value columns of the event spreadsheets.

The speech annotation corpus [2] is organized in subject- and take-specific folders, each containing the associated annotation files (see Fig. 1).

## 4 File Format Specifications

### 4.1 Audiovisual recordings

The audiovisual recordings are stored as MPEG-4 (.mp4) container files with the following technical specifications.

Audio stream

• Codec: AAC (LC)
• Sampling rate: 48 kHz

• Channels: Stereo (2 channels)

• Bitrate: 174 kb/s
• Sample format: Floating Point

Video stream

• Codec: H.264 / AVC
• Pixel Format: YUV 4:2:0 (yuv420p)
• Color Space: BT.709
• Scan Type: Progressive
• Frame rate: 25 fps
• Resolution: 1920 × 1080 (Full HD)
• Display aspect ratio: 16:9
• Sample aspect ratio: 1:1
• Bitrate: 11.3 Mbps

### 4.2 Annotation files

The linguistic annotations are provided in tab-separated value (.tsv) files containing time-resolved event information as described in Table 2.

**Table 2:** Event table description. Linguistic speech annotation variables found in the event file. The methods used to estimate these variables are described in the Experimental design, Materials and Methods section.

Column name (Variable)	Description
onset	Event onset in seconds relative to recording start.
duration	Event duration in seconds.
event_type	The main category of the event. “pause” - onset of a silent interval “word” - onset of a word “phoneme” - onset of a phoneme
mau	Phonetic segmentation of the event in XSampa.
mas	Phonetic segmentation of the event in XSampa including syllable segmentation, the symbol “.” marks syllable boundaries.
kan	Canonical phonological transcript (standard pronunciation) of the event in XSampa.
kas	Canonical phonological transcript (standard pronunciation) of the event in XSampa including syllable segmentation, the symbol “.” marks syllable boundaries.
ort	Orthographic representation of the event.
rftag	Part-of-speech Tags based on RFTagger.
lemma	Base form or dictionary form of the word.
token	Word index starting from 1.

Each annotation folder contains**:**

• *_events.tsv: subject- and take-specific linguistic event annotation file, a spreadsheet that describes the time-resolved linguistic annotation at the word and phoneme level with millisecond resolution.
• *_events.json: subject- and take-specific sidecar file that describes the categorical and value columns of the event spreadsheet.
• *_HED.tsv: subject- and take-specific machine-actionable HED event annotations. **HED** (Hierarchical Event Descriptors) is a framework for annotating events in time-series data using a structured standardised vocabulary [3]. Event descriptions in HED format enable powerful cross-study analysis and work seamlessly with neuroimaging data standards such as BIDS (Brain Imaging Data Structure).

Linguistic units are represented as individual event entries, where each row corresponds to a single annotated event. Depending on the event_type column, events describe pauses, words, or phonemes. Temporal information is represented explicitly through the onset and duration columns, where onset times are given relative to the start of the audiovisual recording and durations relative to the event onset. Additional linguistic information, such as orthographic word forms, phonetic transcriptions in Xsampa, canonical pronunciations, and part-of-speech labels, is provided in the corresponding annotation columns depending on the event type. This event-based representation enables precise temporal alignment across linguistic levels and supports direct integration with event-based neuroimaging workflows and BIDS-compatible analysis pipelines. For this reason, we chose a tabular TSV representation rather than hierarchical annotation formats such as Praat TextGrid or ELAN .eaf.

## 5 Experimental Design, Materials and Methods

### 5.1 Audiovisual recording procedure

The stimuli consist of natural German speech captured in audiovisual recordings of spontaneous, unscripted monologues. Speakers were recruited through a live online casting procedure. Applicants read a short text and spoke freely on a self-chosen topic. A five-member jury evaluated articulation clarity, standard German pronunciation, and voice quality (intelligibility, pleasantness, naturalness). Based on these criteria, six speakers (3 female, 2 male, 1 diverse) were selected for laboratory recordings. In accordance with the participant consent and data-sharing agreement, demographic information is not linked to individual subject identifiers.

During the laboratory sessions, speakers and experimenters were located in separate rooms and communicated via an audio link. Speakers wore open in-ear phones (BatAndCat Sound Labs, Palo Alto, California, US) to hear the experimenter in real time, creating a conversational atmosphere. The experimenter and technical assistance could see the speakers via video monitoring to ensure smooth coordination, encourage natural behaviour, and help maintain gaze toward the camera. Each session began with a short warm-up conversation to establish rapport and to elicit a relaxed speaking style.

Speakers produced free monologues on everyday topics (e.g., hobbies, studies, holidays, part-time jobs). Multiple takes were recorded per speaker. In conditions labelled *"MG"*, continuous cafeteria babble noise [4] was presented at 65 dB SPL(C) through the in-ear phones to elicit Lombard effect; due to the open-fit phones, this level was reached only above 500 Hz. In some recordings, speakers wore glasses (labelled *"BR"*) or applied lipstick (labelled *"LS"*).

Audiovisual recordings were captured with a Canon EOS 700D (Firmware 1.1.4) and a Neumann KM-184 microphone positioned close to the speaker. The camera setup ensured that the speakers’ faces and shoulders were centered and clearly visible. Audio and video were recorded simultaneously, synchronized, and later segmented into shorter “stories” when sessions contained multiple topics. Editing was performed using DaVinci Resolve. Throughout the recordings, video streams were shared with a review panel for visual annotation and selection of the final takes.

### 5.2 Speech annotation pipeline

**Fig. 2:**
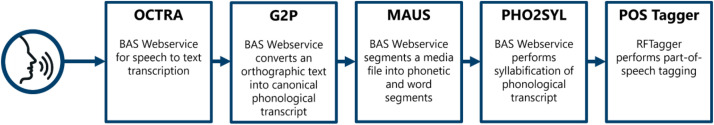
Speech annotation pipeline. Several tools were used in sequence to derive temporally precisely aligned linguistic speech annotations from the audio tracks.

Speech annotation was performed after the audiovisual recordings were collected. The speech annotation pipeline consists of several tools used in sequence (see Fig. 2), including OCTRA, G2P, MAUS, and PHO2SYL, web applications provided by the Bavarian Archive for Speech Signals (BAS) CLARIN center in Munich and were developed for the multilingual processing of spoken language. While OCTRA [5] was used separately, the tools G2P, MAUS, and PHO2SYL were combined in one processing pipeline [6] that performs the different processing steps in sequence automatically. The POS Tagger, a locally installed software from the Center for Information and Language Processing (CIS), LMU Munich, was separately performed at the end of the pipeline to extract part-of-speech tags.

**OCTRA -** The BAS web service Orthographic Transcription (OCTRA [5,7]) was used to perform speech-to-text transcription. Audio tracks were extracted from the original audiovisual recordings [1], saved as separate .wav files, and imported into OCTRA. Automatic Speech Recognition (ASR) was then applied using Google as the provider. The resulting transcripts were saved as text files and manually corrected to ensure that the text is an exact representation of the spoken narrative. This manual correction was essential to minimizing errors in subsequent annotation steps, such as grapheme-to-phoneme conversion and MAUS forced alignment.

**G2P -** The BAS web service G2P [6,8,9] was applied to transform orthographic text into a canonical phonological transcription (KAN) reflecting standard German pronunciation. G2P is trained on an extensive pronunciation database, which it uses to create a language-specific pronunciation dictionary. This tool processes transcripts from the OCTRA step to generate the most likely phoneme sequence. It uses decision-tree algorithms, part-of-speech tagging, and morphological segmentation to enhance accuracy. Phoneme symbols were represented using the X-SAMPA inventory, a standardized, machine-readable format. The process was fully automated, producing text files that list the canonical pronunciation (KAN) of each word in X-SAMPA.

**MAUS –** Then, the Munich Automatic Segmentation (MAUS [6,[10], [11], [12]]) web service was used to analyze both the linguistic and phonetic structures in the audio data. It applies forced alignment to segment continuous speech into discrete word and phoneme units, providing precise start and end times for each segment. The canonical phonological transcript (KAN) from the G2P step is used to estimate the most likely phonetic segmentation and label the speech signal (MAU). To improve temporal resolution, the output frame rate of the MAUS algorithm was set to 1 ms. While not necessarily improving segmental accuracy, it does increase the precision of segment boundary positions, especially beneficial for analyzing phoneme, syllable, and word durations. Additionally, MAUS detects inter-word silence intervals, with the minimal pause length parameter set to 10 ms to identify pauses exceeding this threshold.

The output from MAUS includes a text file containing start times, durations, and both phonetic (MAU) and phonological (KAN) representations of each phoneme in X-SAMPA, along with the orthographic representation of the corresponding word (ORT). It is worth noting that the phonetic (MAU) and phonological (KAN) representations may differ if the speaker deviates from standard pronunciation. In such cases, MAUS aligns and labels the speech based on the actual spoken content (MAU). The output file also includes information about inter-word silence intervals with start times and durations.

To ensure accurate segmentation and labelling, the MAUS output was manually reviewed in PRAAT for forced alignment errors. To further refine segmentation accuracy, speech recordings were manually segmented into smaller intervals. Using PRAAT [13], time intervals (or speech chunks) were defined at the word, phrase, or sentence level. These chunks were then provided as input to the MAUS service, enabling segmentation and labelling within each chunk independently. The resulting outputs from all chunks were subsequently combined into a single file. This manual correction process was repeated twice to minimize alignment errors further and ensure high-quality segmentation.

**PHO2SYL -** The PHO2SYL web service [[6], [9]], part of the BAS toolkit, was applied for syllabification - the process of dividing words into their corresponding syllables. It processes the phonemic (KAN) and phonetic (MAU) transcripts encoded in X-SAMPA and applies a combination of language-specific rules and universal principles, such as sonority ranking, to determine syllable boundaries. The resulting output is a text file that includes syllable segmentation for each word, providing both phonemic (KAS) and phonetic (MAS) representations in X-SAMPA, with boundaries denoted by dots. Like G2P, this step was fully automated.

**POS Tagger –** Part-of-speech (POS) tagging was carried out using the RFTagger, a tool designed to provide detailed POS annotations by Schmid and Laws [14,15]. The RFTagger combines a Hidden Markov Model (HMM) with decision trees. The HMM divides contextual probabilities into products of attribute probabilities and estimates the conditional probabilities. The manually corrected speech-to-text transcript, provided as a text file, served as the input for this process. The RFTagger generates an output text file containing POS tags (RFTAG) alongside dictionary forms (LEMMA) for each word. This processing step was also fully automated.

**Manual correction** – Manual verification and correction were applied at two stages of the speech annotation pipeline, including the speech-to-text transcription (OCTRA) and forced alignment step (MAUS). These manual quality-control procedures were performed by a single trained annotator rather than through independent multi-rater annotation.

## Limitations

One limitation arising in the speech annotation corpus could be related to the annotation pipeline that involved a manual correction step performed by a human annotator. Manual correction by hand suffers from unavoidable interpretation bias and subjective decisions.

## Ethics Statement

The data collection was approved by the local ethics committee (Carl von Ossietzky Universität Oldenburg, Germany, Drs. EK/2021/068) and conforms with the World Medical Association Declaration of Helsinki. All subjects signed written informed consent prior to the data collection and received monetary reimbursement afterwards.

## CRediT Author Statement

**Manuela Jaeger**: Conceptualization; Methodology; Speech data annotation and curation; Writing - original draft; Writing - review & editing; **Mareike Daeglau**: Conceptualization; Funding acquisition; Methodology; Speech data collection and curation; Writing - original draft; Writing - review & editing; **Giso Grimm:** Conceptualization; Funding acquisition; Methodology; Speech data collection and curation; Writing - review & editing; **Martin G. Bleichner**: Conceptualization; Funding acquisition; Methodology; Speech data annotation and curation; Writing - original draft; Writing - review & editing.

## Declaration of generative AI and AI-assisted technologies in the manuscript preparation process

During the preparation of this work the author(s) used ChatGPT 5 in order to improve language and readability of selected sentences. After using this tool/service, the author(s) reviewed and edited the content as needed and take(s) full responsibility for the content of the published article.
